# Investigating the benefits of an arts-based mindfulness program conducted with varsity student athletes

**DOI:** 10.1007/s44192-026-00369-9

**Published:** 2026-02-13

**Authors:** Cole E. Giffin, Diana Coholic, Melonie Gilchrist, Melanie Romain, Thierry R. F. Middleton

**Affiliations:** 1 School of Social Work, Laurentian University, Sudbury, Canada; 2 School of Kinesiology and Health Sciences, Laurentian University, Sudbury, Canada; 3 School of Psychology, Sport, and Health Sciences, Portsmouth University, Portsmouth, UK

**Keywords:** Health promotion, Mental health, Group work, Mindfulness, Arts-based, Varsity athlete

## Abstract

This study explores the benefits of a holistic arts-based mindfulness group program implemented with varsity student athletes through a social group work lens. Recognizing the limitations of individualistic, deficit-based approaches to student athlete mental health, the program integrated experiential arts, group work, and mindfulness practices to promote personal growth and team connection. Grounded in social constructionism and informed by social work principles of strengths-based practice, holism, and relational support, we practiced mental health promotion through arts-based and experiential group activities. Eighteen varsity athletes completed the program, and nine participated in arts-based focus groups to reflect on their experiences within the program. Reflexive thematic analysis was used to create two overarching themes: *Personal Growth: Connecting with Self*, which included increased self-awareness and emotion regulation; and *Team Growth: Connecting with Others*, which reflected improved relationships and mutual aid. The findings suggest that arts-based mindfulness groups offer a promising, interdisciplinary approach to mental health promotion by cultivating increased self-awareness, development of identity, empowerment, and mutual aid.

Mental health is a dynamic state of well-being that allows athletes to realize their potentials, cope with stressors of daily life, work productively, and contribute to those around them [1, 2]. Recently, the mental health of post-secondary student athletes (SAs) has increased within sport and health research [3, 4]. Researchers have suggested that SAs tend to have lower rates of depression [5] and anxiety [6] compared to non-athletes in part due to the positive association between physical activity and well-being. Others have documented problematic behaviours such as drinking and substance use that arise when SAs are unable to cope with their stressors [7, 8]. Such variability can be attributed to research ‘snapshots’ of SA mental health during single time points [3]. SA mental health is temporal and fluid, swaying along the continuum during stressful and less stressful times, such as during exams and peaks in their competitive seasons [4]. The dynamic and contextual nature of SAs’ lives therefore calls for intentional, developmentally informed approaches that strengthen internal and external resources across critical periods of their academic and athletic development [1, 9].

Current research into SAs’ mental health has predominantly centred on pathology, with less attention given to prevention or health promotion [3]. This imbalance can be traced to dominant theoretical framing within the medical model, which typically prioritizes a deficit-based lens and clinical diagnoses over holistic health promotion initiatives [4]. As a result, interventions tend to focus on correcting what SAs lack, often ignoring the broader social, cultural, and systemic contexts that shape their mental well-being. While addressing clinical issues such as depression and acute crisis is crucial and often the focus of professional organizations to bolster performance [10], scholars have argued that individual-level mental health interventions shift the burden of managing stress and distress onto the individual rather than addressing systemic contributors (e.g., pressures related to performance, academic load, organizational culture [11]). Reliance on individualized models obscure organizational responsibilities in promoting mental health, despite the calls for contextualized and systemic mental health promotions within post-secondary settings [8].

## Psychosocial support and group work

The World Health Organization Commission on Social Connection [12]recently identified social health as a key determinant of mental and physical health, emphasizing that mental, physical, and social well-being are deeply interdependent. Lack of social health, often termed isolation or loneliness, can significantly impair SAs’ emotional well-being [13], diminish their self-esteem and sense of belonging [14], and erode trust within teams [15]. Conversely, meaningful social connection with teammates can foster resilience [16], expand psychosocial support networks through reducing stigma around help-seeking [17], and protect against threats to well-being, such identity disclosure regarding triggered by injuries and graduation [18, 19]. Taken together, this body of research underscores the importance for intentional, relationship-centered interventions that strengthen social connection as foundational for SAs’ mental health.

Group work is a social work practice that fosters social connection, mutual aid, and collective empowerment. Grounded in principles of relationality, participation, and shared experience [20,21], social work groups have been reported to offer professional athletes a structured yet flexible environment to explore social connection and mental-health literacy [17]. Through shared strengths and lived experiences, group work positions members as co-creators of healing and change [22]. A supportive group setting can normalize challenges, reduce stigma, and cultivate solidarity among SAs, particularly within sport and academic environments where displays of vulnerability arediscouraged and mental health concerns are stigmatized [23].

### Mindfulness and mental health

Popularized through behavioural medicine, mindfulness, described in the West as “paying attention in a particular way: on purpose, in the present moment, and non-judgmentally” [24, p.145] has grown within the West in several disciplines, including clinical psychology, neuroscience, social work, and sports psychology [25]. Conceptual development of Westernized mindfulness is attributed to clinical approaches that encourage skill development, such as Mindfulness-Based Stress Reduction [24] and Dialectical Behavioural Therapy [26]. With continued practice, like any skill, individuals develop mindful dispositions, whereby life is approached through attitudes of non-judging, patience, beginner’s mind, trust, non-striving, acceptance, and letting go [24]. This conceptualization of mindfulness opens space for considering how these attitudes are cultivated, expressed, and supported within SAs’ everyday environments, and how these attitudes impact SA mental health.

While mindfulness has been traditionally associated with athletic performance [27], growing research demonstrates that mindfulness can benefit SAs’ well-being and performance concurrently, broadly described as thriving [28]. Taught through psycho-educational formats, mindfulness has improved SAs’ emotion regulation [29], stress response [30], non-judgment and compassion of their thoughts and feelings [31], and sense of meaning in life [32]. Mindfulness-based interventions (MBIs) have largely been framed in cognitive and clinical sports psychology, emphasizing individual development through meditation and breathwork. This orientation overlooks the social, relational, and contextual dimensions of SAs’ lived experiences, limiting consideration of how mindfulness might be effectively cultivated within the collective, group-based environments in which SAs train, learn, and compete. There is opportunity to explore alternative approaches to teaching mindfulness in sport, particularly those grounded on social group work that position mindfulness as a personal and relational process embedded in everyday team contexts.

## Mapping the uncharted

Despite important progress in research on SA mental health, key gaps remain. Much of the current literature within intercollegiate sport remains informed by the medical model of mental health [3, 4], resulting in an overemphasis on individualized interventions aimed at crisis response or symptom remediation. This approach overshadows the importance of proactive, strengths-based strategies that foster long-term wellbeing, self-regulation, and supportive sport communities. MBIs are being recognized as promising tools in this proactive space, supporting SAs’ thriving. Framed in cognitive or clinical sport psychology paradigms, these MBIs are administered in groups for logistical ease, but they are not designed to leverage the transformative potential of the group itself. This emphasis on individual change can unintentionally reinforce personal responsibility for mental health, like deficit-based models, while overlooking the relational and social dimensions of positive well-being.

A social group work orientation invites a deeper engagement with the social dynamics of group-based mindfulness and opens space for integrating creative and relational practice, which in turn, can benefit SAs’ well-being through social health and connection. Responding to these gaps, our current research draws on a social group work lens to evaluate a novel, arts-based mindfulness group delivered with varsity SAs. This approach aimed to center relational support and explore how group-based mindfulness promotes SA mental health. This research was guided by the following questions:

1. What were the benefits (if any) of participating in the holistic arts-based mindfulness group?
2. How might this group be used to promote SAs mental health?

## Methodology

This project was grounded in a relativist ontology and social constructionist epistemology. Relativism assumes that reality is multiple and context dependent, shaped through SAs’ lived and relational experiences [33]. Epistemologically, social constructionism holds that knowledge and meaning are co-created through social interaction, language, and experience [34]. In the context of this study, knowledge about the benefits of this program was generated collaboratively with SAs during the program and its evaluation. Accordingly, the methodological focus was on capturing how SAs experienced, interpreted, and made sense of the program. Artistic expression of one’s thoughts, feelings, values, and experiences became a means through which mindfulness was explored, understood, and practiced by way of shared dialogue and collaborative meaning-making. The program and its evaluation were inherently participatory and adaptive, evolving in response to the contributions, insights, and creative processes of the SAs involved. In keeping with this epistemological orientation, we also introduce each author and facilitator to acknowledge our positionality in the development of knowledge.

### Introducing the authors

Author One is a post-doctoral researcher who researches the effectiveness of arts-based mindfulness activities with athletes and elementary school youth. His foundation within performance psychology has situated him as a mental performance coach within an intercollegiate varsity department, where he delivers psychology workshops to varsity teams, including the one in this study. This positioning influenced how he approached the program’s implementation, with, at times, a heavy focus on mindfulness to improve athletes’ performance, consistent with the literature [27]. Connecting with Authors Two, a clinical social worker with over 25 years of experience with arts-based mindfulness modalities, and later, Authors Three and Four, two registered social workers, current graduate students, and experienced facilitators of the mindfulness program with youth populations, resulted in a shift of Author One’s view of mindfulness to a more holistic philosophy, emphasizing both the connection to the self and collective responsibility. Author Two is a pioneer of arts-based mindfulness approaches in social work research and practice. She has extensive research and practice experience drawing on strengths-based group work approaches with youth and adults experiencing mental health concerns. She oversaw the refinement of this program within the varsity setting and provided support when needed. Author Five is a qualitative methodologist with extensive experience with social constructionism and participatory research paradigms within youth sport contexts.

### Introducing the holistic arts-based program

The holistic arts-based program (HAP) has been described in depth in other manuscripts and is available electronically from the Second Author upon request. The program was developed to support for youth living in foster care [35] and has since proved beneficial for youth with challenges in schooling [36], vulnerable children, such as those living off of the welfare system [37], young people aging out of foster care [38], and adults experiencing anxiety and depression [39]. Across all studies, the program has improved participants’ self-awareness, which in turn allowed them practice mindful attitudes, such as non-judgement, to view their circumstances through different perspectives (i.e., flexible thinking) and choose better actions related to their feelings and thoughts [24]. Through a group work approach, participants developed greater social skills, improved coping behaviours, and emotion regulation which allowed improved functioning within their environments [35–39].

The program consists of four interacting, fundamental tenets. First, the program was structured around teaching SAs the core attitudes of mindfulness, as popularized by Jon Kabit-Zinn [24]. Second, the teaching of these tenets took place through experiential arts-based activities. The team was familiar with mindfulness as part of their pre-practice meditation led by the head coach but had not experienced mindfulness or mindful attitudes practiced via different modalities. The variety of activities was important in maintaining a holistic focus towards mindfulness and provided “multiple entry points to practice” [40, p. 10]. Approximately 75 activities, including art, meditation, walking, mindful eating, and group discussions were avenues through which SAs practiced mindfulness. The members performed between five and eight activities per group, depending on the week. Earlier weeks (e.g., one/two) generally included less activities and more focus on group structure, such as group rules. Third, the arts-based activities were conducted using principles of group work, such as group planning, activities and processes to foster socialization and active listening, group agreements, and promoting playfulness [29, 30]. Many activities were conducted in smaller groups within the large group/team (i.e., 4–6 SAs) to foster dialogue and connection, and later, as the group’s connection and cohesion developed, larger group discussion emerged. The group took place in a comfortable room near the SAs’ primary training locations, providing easy access and familiarity.

To illustrate the blending of art, group work, and mindfulness, we briefly introduce one of the first activities taught within the group: the thought jar. In a transparent jar half-filled with water, SAs were invited take a bead, assigning a thought or feeling to the bead, and place the bead in the jar, sharing the thought with the group. This process repeated until each member shared their present moment experience with the group. The jar was sealed and shaken by one of the members. A discussion ensued concerning how we feel when we have many thoughts and feelings swirling around in our minds versus how we feel when our minds are calm, like the jar when the water was settled. We discussed how we can make better decisions when we are in tune and present with our thoughts, noticing them like we notice the beads in the jar.

The final tenet that underpins the program was implementing the activities through a strengths-based approach. Emphasis was directed towards SAs’ “capacities, talents, competencies, possibilities, visions, values, and hopes” [41, p. 297]. An example activity that helps illustrate this approach is called group animal tube. Members were invited to draw themselves as an animal that they feel symbolizes themselves. Emphasis was on aspects of animals that reflected the SAs’ strengths, such as a lion’s bravery and a beaver’s resourcefulness. These animals were glued to a piece of bristol board to symbolize the connection of the team, and members were encouraged to share their animal with the group. This strengths-based approach provided a holistic lens towards internal resources, such as attentional control, resilience, and emotion regulation, that SAs could utilize to promote their mental well-being [4].

### Holistic Arts-Based Program with Student-Athletes

The design and delivery of the HAP was informed by established principles of group work practice (29, 30). Specifically, the program functioned as a planned and purposeful group, with the objective to cultivate SAs’ attitudes of mindfulness, strengthen group cohesion, and foster well-being. The program ran from November 5th, 2023, to February 18th, 2024, consisting of 12, 2-hour sessions per week. These weeks were not consecutive to account for the winter holidays. These sessions were intentionally structured to encourage active member engagement and shared responsibility for the group processes. Each session began with a warmup activity, usually through the form of a game, progressed into one or two main activities, followed with a halfway break including food, one or two more activities, and a closing activity. The program was facilitated by Authors One, Three, and Four. These facilitators met following each session to debrief the team’s functioning and plan the process for the following group.

The program emphasized the development of meaningful relationships and interactions between members and us as facilitators, with attention to creating an environment that respected each SA’s unique needs, experiences, and perspectives. Recognizing that the group was formed of new members, and, judging by our observations, such as hesitancy to share and dependency on the facilitators to lead conversations [30], we located the group within the forming stage of development [42]. Therefore, the beginning of the program was used to establish group rules and foster group interactions, informed by SAs’ opinions of what was needed for a successful group. Examples included a “judgement-free space” and “support.” As the program progressed, group interactions were guided to foster reflection, mutual support, and positive expression of emotions, while maintaining flexibility in group structure and shared leadership to adapt to the evolving needs of the members. For example, session four marked a period where we introduced collective activities that involved group members working together to solve a problem or create a piece of art in which all group members’ identities were represented. As we introduced group activities, we cultivated discussions around the team’s needs and strengths, such as their work ethic, cooperation, and team identity. As the members were empowered to express their individuality, so too was the team, and cohesiveness began to form.

### Participants

The research team obtained research ethics approval prior to the program’s implementation. Informed consent was obtained from all participants in the study.

Author 3 informed participants that the program was being evaluated roughly one month after the last session. Eighteen female SAs participated in the HAP program, and nine volunteered to participate in the post-HAP interviews. While participation in the program was mandated by the head coach, participation in the research was completely voluntary, and members maintained the right to withdraw from the research component of the study at any time. Authors One, Three, and Four presented the SAs with a synopsis of the program and the intended research one week before the program’s implementation. Following the last session on February 18th, SAs were presented with a Google Form where they could indicate their voluntary participation in the program. Athletes who volunteered to participate were contacted by Author One. These SAs were all female, were between the ages of 18–22, represented 50% of the team, and included first year (*n* = 3), second year (*n* = 3), fourth (*n* = 2), and one fifth year athlete. Their sport type is removed for confidentiality. All names found within the manuscript are pseudonyms to protect the SAs’ anonymity.

### Data collection

Author Two engaged in arts-based group interviews as a means of data collection four weeks following the program’s completion. Consistent with our social constructionist positioning, arts-based group interviews fostered cooperation and shared dialogue of the program’s benefits, and it also reflected similar activities found within the program, acting as a memory prompt for the members [43]. Author Two began by encouraging the members to draw their experiences of the program, such as what they like, disliked, or would change. This form of inquiry is conceptually inspired by the work of Neva Boyd [44], a pioneer of play-based recreational movements, who argued that overreliance on verbal expression can constrain meaning-making and learning. Consistent with this perspective, as well as others located within sports psychology research [45], we employed arts-based interviews to foster expression, reflection, and insight that extend beyond purely verbal modes. Members were encouraged to discuss their drawings, and Author Two carefully directed conversation towards aspects of the drawings, such as position on the page, size, relationship with other images, and colors used. Members’ conversations reflected co-developed knowledge between SAs and the interviewer. These three group-based interviews each lasted between 32 and 45 min (m = 39). All group interviews were audio-recorded and transcribed verbatim by Author Three to preserve SAs’ language, tone, and nuances of expression. The resulting transcripts totalled 76 single-spaced of text. Transcription and repeated engagement with the full dataset enabled the research team to attend closely to both content and meaning of members experiences.

### Reflexive thematic analysis

A reflexive thematic analytical process was employed to make sense of the SAs’ experiences and create a shared understanding of the benefits of the HAP program. Drawing on Braun and Clarke’s reflexive thematic analysis [46], our aim was to highlight the patterns of members’ interpretations of the program whilst recognizing our influence in the interpretations of these patterns.

I, Author One, began the analytical process with familiarizing myself with the collected data. I was hesitant that, because I was not present in the interviews, some of the inherent meanings, such as members’ tone, body language, and facial expressions, that provide context for meaning would be missing from the analysis. I began with listening to the interview recordings, meeting with Author Two and Three to discuss the interviews and returned to my logbook to reflect on my experiences within the group. During this phase of the analysis, I was excited that I was studying mindfulness in a sports context. Mindfulness practice formed a major part of my life following my transition from university sport, and I was inspired to share mindful activities with undergraduate SAs, an opportunity I wished was present during my schooling.

As I iteratively moved between the data sets, I deductively and inductively coded the data through the lens of mindfulness attitudes [24] and psycho-social benefits of the program, including development of identity, improved relationships, and reduced stress [35–39]. I was careful during this phase, knowing that my experiences with mindfulness might cause me to favor the positive benefits of the program. Monthly meetings with Author Two, a critical friend [33], challenged my assumptions and tied the findings back to important indicators of positive mental health and group challenges. During the time of this analysis, I was also working with additional varsity teams in my capacity as a mental performance consultant. I frequently employed a variation of mindful awareness, titled reflexive self-consciousness [14], to help student-athletes become mindful of their sport and academic related goals. This type of awareness differs from Kabat-Zinn’s attitudes of mindfulness in that reflexive self-consciousness relates to controlling one’s attention (e.g., placing attention on a personal or team goal), while the program’s mindfulness was rooted in observing thoughts without control [24]. When analyzing, I tended to link findings back to performance psychology, viewing mindfulness as a benefiter and controller linked to performance. Author Two challenged this interpretation through drawing my attention towards SAs’ abilities to observe and not create their experiences, such as their feelings during activities that were emotionally intense.

Codes were grouped into themes to represent our interpretations of patterns within the data. Given that social group work is often an iterative, mutual process that benefits the individual and the collective [31], we grouped the codes and sub-themes under two overarching themes of individual and group benefits. While this decision might initially come off as “domain summaries” [46, p. 592] consistent with codebook forms of thematic analysis, they were intentionally chosen to reflect the dyadic nature of group work. We defined each theme, created a thematic table, and produced the final report.

### Quality

We assessed the quality of this research through characteristics relative to our social constructionist position. Aligned with the epistemological positioning outlined in the methodology, we recognize that truth is not viewed as objective but instead was co-constructed between group members and our research team. Themes and quotations to support the themes dynamically shifted throughout the research project.

In aiming to align these findings to the participants’ perspectives and enhance authenticity, we utilized a member reflections technique [33] with five willing group members to invite dialogue and generate further insight into the nature of the findings. Author One shared the thematic table and a copy of the results with the willing members. One reflection shared from three of the participants was the need to include more quotations relating to the personal transformation that occurred within the program. Members wanted to highlight the fact that these internal transformations allowed members to bring their best selves to the group, and that personal growth is one factor that led to team growth.

We also maintained quality through practicing transparency and reflexivity throughout the manuscript. In keeping with the relational commitments of social constructionist inquiry, we approached this process with humility, openness, and transparency, attending to how our authors shaped the knowledge produced [34]. Layered throughout the methodology are reflexive accounts of our influence within the research process, including our roles, interpretations, and interactions with participants. In addition, we sought to foreground multiplicity and contextual nuance in our presentation of the findings, offering space for diverse interpretations and resisting simplistic or universal claims.

## Results

The purpose of this project was to explore the benefits of HAP from the perspectives of SAs and connect these benefits to mental health promotion. Two overarching themes were developed from the analysis, each containing relevant sub-themes. The first theme, *Personal Growth: Connecting with Self*, captures how SAs individually benefitted from the program. The second theme, *Team Growth: Connecting with Others*, reflects how individual growth contributed to stronger group dynamics and collective cohesion.

### Personal growth: connecting with self

This theme highlights the internal transformations SAs experienced as a result of the program. Two sub-themes illustrate these changes: *Increased Awareness* and *Emotion Regulation*. Together, they reflect how HAP supported SAs in developing deeper self-understanding.

#### Increased awareness

Members engaged in over 60 experiential, arts-based mindfulness activities designed to cultivate attention to the present moment. Initially, awareness was externally directed towards sensory stimuli of SAs’ surroundings. Harper explained, “When we listened to the music or we had to draw something or when we went on a walk, like looking at things… it broadened my understanding of mindfulness.” Similarly, Parker noted how the program helped her develop a mindful attention to her environment: “Noticing a color when you’re walking outside. Being present, I think that helped me notice the things around me.”

Seemingly simple practices, such as mindful walking, laid the foundation for more meaningful awareness that developed over the course of the program, coinciding with introspective, reflective activities. Members noticed fleeting emotions, thought patterns, and assumptions about themselves, many of which reflected internalized expectations associated with their athlete identities, such as performance-related self-evaluation and self-criticism. For example, Rowan said “I learned a lot about myself. I replayed the mistakes I made, and I felt bad. My mind always goes to [sport].” This heightened attention to sport performance illustrates how a salient athlete identity can shape patterns of attention, even during arts-based mindfulness activities. However, other athletes, like Aiden, described this shift in self as a relief “Being in, like, an environment where we could express ourselves and not have it be [sport]-focused was nice.” While the word “nice” minimizes the weight of this moment, her statement points to a larger process of reclaiming her selfhood beyond her athletic identity. Aiden later stated that the program “helped with identity. Because I feel like we associate ourselves as [athletes] all the time.”

Such reclamation of identity encouraged members to reconnect with parts of themselves that had been overshadowed by performance-driven culture. Cameron noted discovering the “courage to share in front of a group,” a skill not typically emphasized in sport contexts. Likewise, for Avery, the activities prompted emotional reflection and rediscovery of activities she liked to do as a child, somehow forgotten during the progression through university:

Going back to art and stuff that we used to do as kids and [pause], who I am. I know more of who I am now… it got me to reflect on like, ‘Oh, I think I wanna touch more on this. I really am happy about this.

Her words suggest that the arts-based practices not only facilitated emotional insight of things that she enjoys but rekindled a sense of personal meaning.

As members’ awareness expanded, some SAs also recognized how they could actively care for their well-being and cope with life demands. Parker explained that creative activities helped her decompress from stressful events in her life: “I needed creative activities to not think about everything else going on in life.” This ability to mentally shift away from chronic stressors (e.g., academic deadlines, sport performance) was critical for sustaining mental health in high-pressure environments. Indeed, some SAs realized that performance was not the end all priority, and that balancing it with well-being is a necessary part of being human. As Avery put it succinctly, the program reminded her that “we are all people, not just athletes,” an important re-humanizing realization in a culture that often prioritizes output over wholeness.

#### Emotion regulation

Greater self-awareness fostered members’ abilities to regulate their emotions. SAs consistently described how the mindfulness activities helped them downshift from stress and re-engage with their emotions in more adaptive ways. The group sessions became a space for emotional rest, one that contrasted sharply with the academic and athletic pressures they carried into the room. Cameron shared

I’d walk in [the group room] and be like stressing about like something that I had to study for or an assignment I could have been doing. Once we started going, I would like completely forget about it. Which was so nice. So, like have that kind of balance between like oh, stressing about [sport], and oh stressing about school and then when you’re there, you kind of are just like focusing on what you’re doing.

The quote above illustrates how the arts-based mindfulness activities acted as a buffer against cycles of rumination and shifted members’ attention back to the present. Importantly, these were not one-size-fits-all techniques. The variety of modalities allowed athletes to experiment and personalize their regulation strategies. Parker found value in meditation: “It opened my mind to self-control and it calmed me down.” Meanwhile, Harper gravitated toward art-based expression: “Mindfulness isn’t just meditation… [Art] was easier to communicate things. [It] also makes it easier to talk about it.” These insights underscore how the program honored different learning and coping styles. For some, stillness and introspection helped regulate; for others, creativity and expression helped.

Members also applied these skills in real life, signaling a transfer of learning from the group to the broader world. Aiden used grounding techniques at the end of her day to reflect without judgment. For Rowan, breathing had a significant impact on performance anxiety related to her sport:

I would just throw up from being so nervous… Then one game, I was starting to feel my stomach, you know, and like those feelings before. So I went and literally just did a breathing exercise… just being completely present and not worrying… Like my life’s not going to end because I am nervous about this game.

Rowan’s experience demonstrates how mindfulness helped reframe high-pressure moments. The practices did not eliminate anxiety, but they empowered her to respond differently and make better decisions, shifting from catastrophizing to acceptance. Her reflection also points to a deeper ability to discern what was in her control and to let go of what was not.

### Team growth: connecting with others

This theme captures the social and emotional transformations experienced by SAs through their participation in the HAP program. This overarching theme includes two sub-themes: *Building Relationships* and *Mutual Aid*. They illustrate how the program facilitated cohesive, empathic, and collaborative team dynamics by disrupting patterns of judgement and isolation.

#### Building relationships

Before the program, many athletes described the team environment as fragmented, emotionally distant, and judgmental. Cameron, for example, recounted how initial group norms around non-judgment were undermined by sarcasm and banter, which discouraged her from openly sharing her experiences with the group. Similarly, Avery “never felt comfortable during her first two years on the team”, and Riley observed that the team dynamic was “rooted in separation.” Indeed, these unspoken cultural norms within this varsity environment undermined authentic social connection between teammates.

Such dynamics are not uncommon in varsity sports contexts, where structural barriers, like constant turnover, inhibit authentic relationship building. Indeed, Parker, a fourth-year veteran of the team, described the difficulty navigating relationships because of “new people coming in every year and people leaving every year… All we see is [each other] as a teammate. We don’t get to know like, who they actually are.” This constant turnover means teammates were often reduced to their athletic identities, perceived through the lens of performance rather than personhood.

As the HAP program unfolded, however, these relational patterns began to shift. Through shared artistic expression and storytelling, athletes began to connect in ways that transcended their athletic identities. Cameron enjoyed how “not everything had to relate back to [my sport],” because “you can actually like learn who people are outside of [sport],” which allowed for a humanizing of teammates. Indeed, creating an “opening to [learn] who they really are” (Avery) and “connecting as people and not as a team” (Remi) was influential for establishing relationships between teammates. This non-performance-based context created space for vulnerability, which opened the door to authentic connection, belonging, and group cohesion.

Small but meaningful shifts in connection, such as noticing teammates’ facial expressions or adapting communication styles, became indicators of deeper relational awareness. Remi reflected on how these subtle cues became tools for emotional support and more effective on-court communication, “being able to talk to them in a way that they can understand or in a way that they can feel appreciated still was a game changer.” This process of trust, non-judgment, and socialization was important for all athletes, but was continually reinforced by the first year SAs, like Harper, who emphasized how these experiences helped her feel welcomed and known, disrupting the typical disorientation that comes with entering a new competitive environment:

But like you got, yeah, you got to know people more, like outside of [sport]. And just more about like their personal lives and stuff. So like that was good too, especially just like coming in and like being new. You don’t have a lot of time or maybe didn’t have enough time yet to get to know each other.

#### Mutual aid

As SAs’ relationships with each other deepened, the group dynamic evolved toward mutual aid, a reciprocal emotional support and collective responsibility for helping one another. Athletes shared more openly about both lighthearted and serious aspects of their lives. For instance, Riley shared her enjoyment of laughter which became a bonding agent, while more vulnerable disclosures were met with empathy rather than judgment. Art acted as a conduit for emotional expression. For some, like Quinn, the opportunity to “speak and be heard” when sharing one’s art.

offered healing from difficult times in her life. For others, like Rowan and Remi, the visual and symbolic language of painting allowed them to perceive teammates’ emotions in new and meaningful ways. Rowan noted how she could “tell how everyone was feeling that day because of the painting,” signaling the development of emotional attunement within the team.

These interpersonal shifts also nurtured compassion. Parker, who initially found it hard to open up to her teammates, explained that the program helped her “learn about [her]self, which helped [her] find more compassion for [her] teammates and connect with [her]teammates in a different sense than [sport].” Compassion became an embodied form of emotional intelligence expressed through listening, attunement, and checking in on others during challenging moments.

This emotional resonance had a ripple effect. Mel noted how understanding a teammate’s difficult day changed her approach in practice, motivating her to “lift them up more,” in times of need. These acts reflect a growing ethic of care within the team, a contrast to the individualistic and performance-oriented culture in sport seen at the beginning of this second theme. The final outcome was the team’s movement toward collective action. The team intentionally used practices from the HAP program to shape team culture and reconstruct important events in the team’s development. One such example was the use of the Thought Jar, a check-in activity that became a pre- and post-game ritual. This not only helped athletes gauge each other’s emotional states but also set a tone of mutual support and shared intention before competition. Even after losses, the SAs used these tools to process disappointment and regain perspective. As Remi described, practices like reflective team discussion helped her emotionally decompress and “put things in perspective,” supporting her mental health in a high-pressure environment.

## Discussion

We explored the benefits of HAP from the perspectives of varsity SAs. Two overarching themes were created: *Personal Growth: Connecting with Self* and *Team Growth: Connecting with Others*. These themes illustrate how arts-based mindfulness practices supported SAs’ mental health and catalyzed a shift in the team’s social dynamics. The manuscript situates SAs’ mental health within a health promotion context and bridges social work-related concepts, such as mutual aid and strengths-based practice, with sports psychology. This discussion is used to interpret these findings in relation to existing sports psychology and social work literature, draw links between the results and health promotion initiatives, and outline limitations and directions for future research in relation to SAs’ mental health.

The first finding highlights SAs’ internal transformations via self-awareness and emotion regulation. The progression from external, sensory-based awareness (e.g., noticing colors or music) to deeper self-reflection suggests a broadening of attentional focus, in line with the goals of mindfulness practice in sport [24, 27]. SAs noticed their environments, emotions, identities, and thought patterns, often paying attention to these non-judgmentally for the first time. For some, this was a sudden and shocking revelation, aligning with research on athlete identity foreclosure, which suggests that over-identification with sport hinders broader identity development [18]. Through reflective art-making and non-sport-related activities, athletes reconnected with parts of themselves previously overshadowed by competitive culture and striving for sport success, suggesting identity expansion. Engaging and exploring multiple parts of one’s identity is not only important for optimal human development [47] but has been shown to mitigate aspects of psychological distress, helps SAs cope with stress, and create a sense of self-worth by reducing SAs’ over-identification with performance outcomes; a key risk factor for depression and burnout [48]. Sport MBIs should be designed to deliberately foster identity expansion, not merely attentional control or performance regulation, like reflexive self-consciousness [26]. Guiding SAs from externally focused awareness toward mindful attitudes on emotions, thoughts, and identities may help disrupt identity foreclosure and reduce over-identification with performance outcomes, supporting psychological well-being and protecting against burnout and distress.

Emotion regulation emerged from identity expansion as a related sub-theme. Consistent with research on the positive relationship between emotion regulation and mental well-being [49], arts-based mindfulness practices helped SAs manage stress, reduce rumination, and shift into adaptive emotional states. Importantly, the creative, strengths-based approach taken towards fostering emotion regulation within this project adds to this cognitive and clinical psychology dominated space. While effective, cognitive reappraisal techniques to emotion regulation are pursued through a lens of control; an SA feels an emotion and activity tries to mitigate their response. Alternative theoretical perspectives, such as Mindfulness-Acceptance-Commitment (MAC) approaches [50], challenge the control narrative by highlighting that thoughts and emotion happen to us and acceptance of, rather than control over, these emotions are imperative. The MAC approach also highlights that acceptance is not just a cognitive exercise but needs to occur experientially. Creative, and strengths-based practices offer utility towards approach experiential strategies for emotion regulation [51], particularly because the embodied act of practicing art integrates the physical, emotional, and cognitive aspects of the self [52]. Perceiving oneself as strong, capable, and confident increases one’s ability to regulate emotions [51] and fosters a sense of empowerment needed for SAs to devise ways to address their own needs. This variation to emotion regulation underscores the value of a multimodal approach to mindfulness, which supports diverse coping styles and emotional needs of SAs. The transfer of these practices into pressured competition settings further highlights their real-world utility for incorporating mindfulness into sports contexts, consistent with current literature on mindfulness and thriving [28]. Arts-based mindfulness activities offer simple, cost-effective, and strengths-focused and embodied pathways through which student-athletes notice and accept emotions as they arise, rather than manage, control, or suppress them.

Consistent with health promotion and social group work literature, wherein a healthy individual can help transform the group, and vice versa [20], the program fostered meaningful social transformations within the team. Members described the pre-group team culture as disconnected and fragmented, shaped by unspoken norms of competition and judgment. These norms made it difficult for members to relate outside of sport and reinforced their athletic identities as they tried to best their teammates. These findings reflect broader patterns in varsity sport, where frequent turnover and performance-oriented culture inhibit trust and cohesion and impact SAs’ mental health through performance pressure [30]. The program disrupted these pre-existing dynamics by introducing non-performance-based forms of connection through group-based activities. As athletes shared stories, created art, and engaged in emotional reflection, they related to each other, demonstrating empathy and normalizing vulnerable experiences. This shift from judgment to empathy was made possible through group-based creative experiences that allowed for emotional risk-taking in a safe and supportive environment. These relational shifts mirror constructs of psychological safety and interpersonal trust, both critical for effective teamwork and health promotion [53].

Without such a relational foundation, the sub-theme of mutual aid would not be possible. Mutual aid illustrated how this emotional closeness translated into tangible acts of care and compassion. SAs began checking in on each other, adapting their communication, and offering support during difficult moments. While this finding might, on its surface, reflect the large body of sports psychology literature on the benefit of positive social support within teams [16, 48], we interpret it bi-directionally. Social support within sport is often one-directional, with expert knowers like sports psychology practitioners, coaches, older mentors supporting the SAs in need. However, support, or mutual aid in social work terms, occurs reciprocally between members [22]. Members became competent in giving and receiving help from others as needed, creating “many helping relationships” [22, p. 19]. This finding bears similarities with Peterson and colleague’s [54] constellation mentoring program, where mentoring occurred in groups as opposed to single mentor-mentee dyads. The act of helping teammates strengthened SAs’ ability to learn to help, which with practice, can be internalized into helping oneself, fostering a deeper form of empowerment [55]. Moreover, mutual aid is inherently holistic [55], and SAs brought the best of themselves, including their skills, capabilities, and potentials, to help members and the group flourish. Mutual aid within teams fosters a more egalitarian, reciprocal form of support, which differs from traditional hierarchical models discussed in sports psychology. This in turn creates a community of support and a belief that SAs have the capacities and resilience to deal with and promote each other’s mental health and well-being.

### Applied implications

SAs routinely navigate high cognitive, emotional, and physical loads associated with their dual careers [3, 4], and difficulties with stress management, emotional regulation, and psychological well-being are known to impair both academic functioning and athletic performance [8]. The present findings have applied relevance beyond mental health alone, as improvements in emotion regulation, stress management, self-awareness, and social support directly support performance-related capacities such as focus, anxiety, resilience, and recovery. By addressing the psychological and relational foundations of well-being, the intervention contributes to conditions that enable SAs to more effectively balance academic responsibilities with athletic performance, an essential requirement for thriving [28].

The findings of this study therefore offer several practical implications for coaches and sport psychology consultants (SPCs) seeking to support both SAs’ well-being and performance. First, the results highlight the value of integrating group- and arts-based mindfulness activities into training and team development programs as a complement to traditional performance-focused interventions. Coaches and SPCs can incorporate brief creative and reflective exercises, such as visual journaling, metaphor drawing, group storytelling, and guided imagery paired with artistic expression, to cultivate safe spaces that encourage awareness, reflection, and mutual aid (interested readers may contact Author Two for sample activities).

Second, the relational structure of the program underscores the importance of fostering team environments that normalize vulnerability and support collective meaning-making. Coaches and SPCs can enhance team cohesion and emotional regulation by facilitating structured group discussions and creative activities that allow SAs to express and process their experiences beyond performance outcomes. For SPCs, this approach provides an accessible framework for promoting mindfulness as both an individual and relational resource, especially for SAs who struggle with traditional meditation-based interventions while managing dual career demands.

Finally, these findings suggest that well-being initiatives in sport are most effective when embedded within broader team culture rather than delivered as isolated mental skills sessions. Integrating relational and arts-based mindfulness practices into everyday environments can strengthen emotional regulation, resilience, and social support. These psychological capacities can sustain both personal well-being and long-term performance.

### Limitations and future directions

This study is not without limitations. The sample size was relatively small and drawn from a single varsity team, which limits generalizability to other sports, genders, and university contexts. Future research might consider implementing and evaluating across different genders, sports, and competitive levels to understand how the program might become adaptable to diverse athlete populations. Second, the study did not include a follow-up component, so the durability of changes over time remains unknown. Longitudinal designs could assess whether the personal and team-level shifts observed here are sustained over time. Finally, while the qualitative nature of this paper was intentional given the novelty of group and arts-based practices within sports, researchers could also examine the specific mechanisms by which art and mindfulness interact to produce emotional insight and social connection, perhaps using mixed methods research approaches.

A further limitation was the limited attention to cultural and social factors that shape how mindfulness, mental health, and arts-based practices were experienced. Although the program emphasized relational and group-based engagement, the study did not explicitly examine how gender, race, sexuality, sport, or other social identities influenced members’ comfort with mindfulness practices, emotional expression, or engagement in creative, group activities. As Kirmayer [56] explained, meditative technologies, arts-based mindfulness included, cultivate and configure subjectivity, social identity, personhood, and ways of being. Mindfulness reflects culturally mediated forms of understanding conveyed through art, and cultural nuances convey different understandings, effectiveness, and lived experiences. For example, mindfulness and arts-based approaches may be received differently within men’s teams, where norms of masculinity can discourage vulnerability or emotional disclosure [57]. Similarly, the absence of discussion regarding racial and cultural backgrounds risks positioning SAs as a homogeneous group, despite understanding that social identities and structural inequities shape experiences of sport participation and mental health. With this said, future research would benefit from exploring how intersectional identities and cultural contexts interact with mindfulness interventions. Such work could explore how arts-based and relational approaches could be adapted to reflect diverse ways of knowing and expressing emotion, thereby strengthening the inclusivity and relevance of mental-health promotion in sport.

## Conclusion

This study highlights how a holistic arts-based mindfulness program functioned as a proactive health promotion initiative in a varsity sport context. By shifting away from deficit-based models of mental health, the program created a space for SAs to explore mindfulness as a relational, strengths-based, and creative process, specifically emphasizing self-discovery and social connection. The program increased SAs’ self-awareness, emotion regulation, and identity expansions, and strengthened interpersonal dimensions of well-being by promoting mutual aid, psychological safety, and social connectedness among teammates. Integrating principles from social work and sport psychology, this research exemplifies how creative, group-based mental health interventions can promote mental health and well-being. As such, this study advances the conversation on health promotion in sport by offering a relational and arts-informed approach that centers individual thriving and collective well-being.

## Data Availability

The datasets generated during and/or analysed during the current study are available from the corresponding author on reasonable request.
